# A Review of Subsurface Electrical Conductivity Anomalies in Magnetotelluric Imaging

**DOI:** 10.3390/s23041803

**Published:** 2023-02-06

**Authors:** Wule Lin, Bo Yang, Bo Han, Xiangyun Hu

**Affiliations:** 1 Hubei Subsurface Multi-Scale Imaging Key Laboratory, School of Geophysics and Geomatics, China University of Geosciences, Wuhan 430074, China; 2 Key Laboratory of Ocean and Marginal Sea Geology, South China Sea Institute of Oceanology, Innovation Academy of South China Sea Ecology and Environmental Engineering, Chinese Academy of Sciences, Guangzhou 511458, China; 3 Southern Marine Science and Engineering Guangdong Laboratory, Guangzhou 511458, China; 4 China-Pakistan Joint Research Center on Earth Sciences, CAS-HEC, Islamabad 45320, Pakistan; 5 Institute of Geological Survey, China University of Geosciences, Wuhan 430074, China

**Keywords:** magnetotelluric, electrical conductivity, fluids, partial melt, graphite films, sulfide, water content

## Abstract

After 70 years of development, magnetotelluric (MT), a remote sensing technique for subsurface electrical resistivity imaging, has been widely applied in resource exploration and the deep tectonic evolution of the Earth. The electrical resistivity anomalies and their quantitative interpretation are closely related to or even controlled by the interconnected high-conductivity phases, which are frequently associated with tectonic activity. Based on representative electrical resistivity studies mainly of the deep crust and mantle, we reviewed principal electrical conduction mechanisms, generally used conductivity mixing models, and potential causes of high-conductivity including the saline fluid, partial melting, graphite, sulfide, and hydrogen in nominally anhydrous minerals, and the general methods to infer the water content of the upper mantle through electrical anomaly revealed by MT.

## 1. Introduction

**Figure 1:**
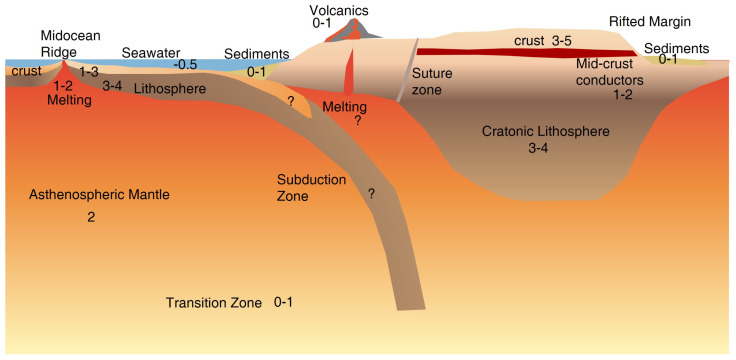
A cartoon showing key features of crust and upper mantle and their typical ranges of resistivity with numbers denoting resistivity in $\log_{10}\left(\Omega .m\right)$ as summarized by Evans [5].

Magnetotelluric (MT) imaging is a geophysical method for imaging the electrical resistivity (or its reciprocal conductivity) of the Earth’s interior from the surface to the mantle transition zone based on the simultaneous measurements of the horizontal components of time-varying natural electromagnetic (EM) fields on Earth’s surface [1,2]. In a uniformly half-space medium, the penetration depth of EM fields is described by skin depth $\sigma =503\sqrt{\rho T}$, where ρ denotes the apparent resistivity that depends on Earth’s resistivity structure (Figure 1) and T means the period of the EM field variations of MT at ranges of $10^{-4}–10^{5}\;s$. The fundamental theory of MT was proposed independently by Tikhonov [3] and in more detail by Cagniard [4]. Both of them realized that EM responses from great depth could be obtained by extending the MT period as described in skin depth [1].

Since then, several technique revolutions greatly promoted the development of MT and its applications, mainly including the robust estimation of MT response functions e.g., [6,7], the realization and removal of the galvanic distortions of MT data due to near-surface inhomogeneities e.g., [8,9,10], developments in two-dimensional (2D) and three-dimensional (3D) MT inversion with electrical isotropy or anisotropy e.g., [11,12,13,14], and the electrical measurements of major minerals in the crust and mantle in the laboratory at high temperatures and pressures e.g., [15,16,17].

Benefiting from these technical improvements, MT has been widely used in various fields, such as geothermal prospecting [18,19,20], mineral exploration [21,22,23], volcano studies [24,25,26], earthquake generation studies [27,28,29], studies of structures and processes in the continental crust and mantle [30,31,32,33,34]) and marine surveys [35,36,37]. With the development of the new MT instrument [38], MT probably would be used to probe the interiors of other worlds.

Electrical conductivity varies over many orders of magnitude (Figure 1), making it suitable to differentiate Earth’s materials especially when considering the geological setting, tectonic activity and other information. In particular, the interconnected conductive materials, often closely related to the tectonic processes generally control the bulk resistivity imaged by MT. These primary conductive phases are saline fluids, partial melts, graphite, sulfides and water (hydrogen) in nominally anhydrous minerals(NAMs). This paper presents a systematic review of the state of the art in research on high conductivity anomalies related to MT by reviewing the conduction mechanisms of crust-mantle rocks, the generally used mixing models, the potential causes of high conductivity anomalies by each conductive phase, and the general methods to infer the water content of the upper mantle.

## 2. Magnetotelluric Method

MT is a passive electromagnetic (EM) method for probing the subsurface electrical resistivity structure of the Earth [1]. The information on the subsurface electrical structure contained in MT data is derived from the simultaneous measurements of natural time varying electric (E) and magnetic (H) fields in the orthogonal horizontal direction on Earth’s surface. After transforming to the frequency domain, the E and H fields can be linked via a complex impedance tensor Z as follows [1]:

(1) $$\left(\begin{array}{c} E_{x}\\ E_{y} \end{array}\right)=\left(\begin{array}{cc} Z_{xx} & Z_{xy}\\ Z_{yx} & Z_{yy} \end{array}\right)\left(\begin{array}{c} H_{x}\\ H_{y} \end{array}\right),$$

The usually used apparent resistivity $\rho_{a}$ and phase ϕ can be derived directly from the complex impedance tensor:

(2) $$\begin{array}{c} \rho_{a}=\frac{1}{\omega \mu_{0}}{\left|Z\right|}^{2}, \end{array}$$

(3) $$\begin{array}{c} \phi =\tan^{-1}\frac{imag(Z)}{real(Z)}, \end{array}$$

where ω is the angular frequency and $\mu_{0}$ is the permeability of free space.

## 3. Conduction Mechanisms

Electrical conductivity (σ) is related to the transmission of electrical currents by free charge carriers [1], and can be described for a specific charge carrier as follows:

(4) σ=nqμ

where n, q, and μ separately represent the number, charge and mobility of the charge carrier. The conductivity of minerals or rocks may be composed of several conduction mechanisms and thus is the sum of all of them. There are three primary conduction mechanisms in the minerals and rocks of the crust and upper mantle.

### 3.1. Electronic and Ionic Conduction

The classic examples of electronic conduction are metallic ore minerals and graphite, where the free electrons (charge carriers) are not firmly bound to atoms and can transport charge, making the metallic ore highly conductive compared with the generally resistive host rock. MT is hence widely successfully used in mineral exploration such as the largest volcanic-related uranium deposits in China [23] and one of the world-class magmatic mineral systems in Australia [21]. Unlike metallic ore minerals, a small number of graphite films (with thickness <100nm) would increase the bulk conductivity dramatically as long as the graphite films on mineral grain boundaries are interconnected [39,40]. However, the evidence for interconnected graphite films over large scales is limited [5].

Ionic (or electrolytic) conduction generally occurs in fluids containing dissolved free ions (e.g., Na${}^{+}$) that can easily move. As shown in Equation (4), the conductivity of the fluids is determined by the concentration, charge, and mobility of charge carriers, provided they are interconnected such as in the pore space. In addition, partial melting occurs commonly in tectonically active regions and also acts as ionic conduction [1]. Therefore, MT can be used to constrain the pore distributions, the porosity of rocks, and partial melt fractions, combining the mixed model in Section 4.

As shown above, conductive phases with trace amounts if interconnected would be able to raise the bulk conductivity [2,41], and hence become the target of MT. More details of each conductive phase will be discussed in Section 5. Ignoring these conductive impurities, the semi-conduction would be the major conduction mechanism in the mantle.

### 3.2. Semi-Conduction

Temperature is one of the principal factors in controlling the mobility of charge carriers and thus the conductivity of materials [5]. Earth’s crust and the upper mantle are generally composed of silicate minerals, which behave as insulators near the surface; at greater depth (e.g., the lower crust or mantle) high temperatures would make these minerals behave as semiconductors [40]. Hence semi-conduction is expected to be a dominant process in mantle minerals [1]. The conductivity of silicate minerals can be defined using the Arrhenius equation [42]:

(5) $$\sigma =\sigma_{0}\cdot exp\left(\frac{-\Delta H}{RT}\right)$$

where $\sigma_{0}$ is the pre-exponential factor, ΔH is the activation enthalpy, *R* is the gas constant and *T* is temperature.

**Figure 2:**
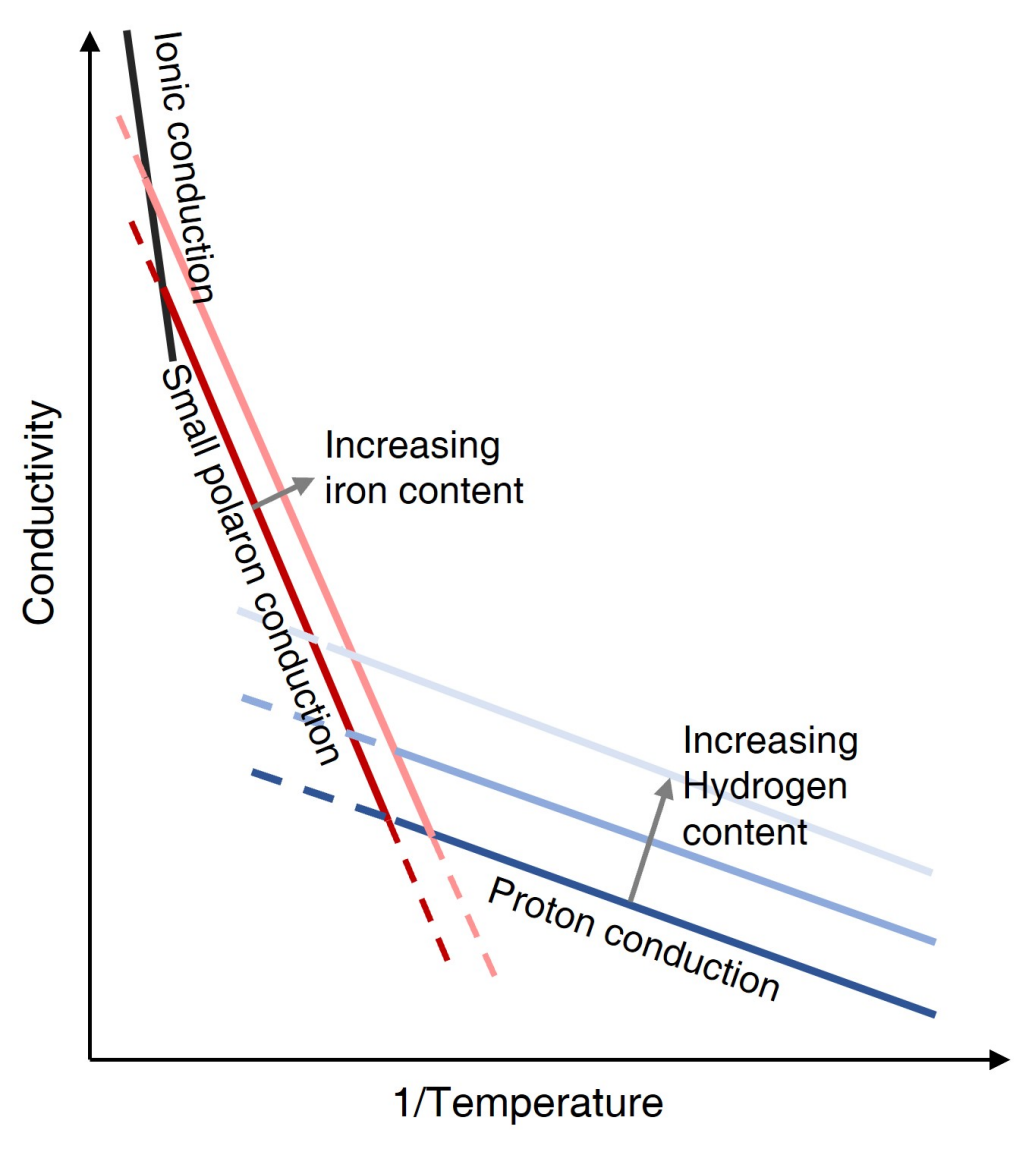
The change of silicate minerals conduction mechanism with temperature [40]. The specific temperature and conductivity is compositionally dependent.

As shown in Figure 2, there are three main conduction mechanisms for the silicate minerals at high temperatures. The dominant conduction mechanism changed from proton conduction ($H^{+}$) to small polaron conduction (Section 3.2.1) to ionic conduction with increasing temperature. The conductivity of a material is the sum of all possible conduction mechanisms: $\sigma =\sigma_{pro}+\sigma_{pol}+\sigma_{ion}$, where $\sigma_{pro}$, $\sigma_{pol}$ and $\sigma_{ion}$ are the conductivity of proton, small polaron and ionic conduction, respectively. Note that ionic conduction occurs at extremely high temperatures close to the melting point [43]; for instance, for olivine (the major mineral within the upper mantle), it occurs at ∼1300 °C through the creation of cation vacancies in magnesium or iron site [40,43,44,45].

#### 3.2.1. Small Polaron Conduction

Small polaron conduction occurs through electron hopping or diffusion between ferrous (Fe${}^{2+}$) and ferric (Fe${}^{3+}$) iron, and is proportional to the iron or Fe${}^{3+}$ content (Figure 2) apart from temperature [40,43,46]. The increasing iron content could decrease the distance between ferrous and ferric iron, and hence probably decrease the activation enthalpy required by small polaron conduction [43]. However, the differences in iron content among peridotite are generally small [47], resulting in a generally negligible effect of the iron content on small polaron conduction [48]. In comparison, the iron content between the crust and mantle is largely different, resulting in a change in conductivity of up to over an order of magnitude [49]. Therefore, it would be better to take the iron content into consideration particularly when studying the resistivity of the crust and mantle together. Moreover, the conductivity of polaron conduction is influenced by oxygen fugacity as it is proportional to the ferric iron content [43].

The theory of small polaron conduction is well reviewed by Yoshino [43]. In general, for the main minerals in the upper mantle, the influence of composition on the conductivity is less than a quarter of an order of magnitude, while that of the oxygen fugacity is less than half of an order of magnitude, and the influence of temperature (for the lithosphere mantle) is larger than three order of magnitude [50]. Even though the effects of composition and oxygen fugacity on conductivity are relatively small compared with temperature, considering as many factors as possible would give a more reliable upper mantle temperature estimation using MT studies [51].

#### 3.2.2. Proton Conduction

The nominally anhydrous minerals (NAMs) (e.g., olivine, pyroxene, garnet and plagioclase), which make up the majority of the bulk mantle can store water as the hydrogen species in the form of hydroxyl (OH${}^{-}$) within their intrinsic point defects [43]. Hydrogen (water) is expected to have large solubility and mobility in NAMs of solid-state and hence proton conduction can occur through hydrogen diffusion among point defects [52]. Various laboratory measurements at high temperatures and high pressure have demonstrated that increasing the water content would enhance the electrical conductivity of NAMs [53,54,55,56] as first proposed by Karato [52], which however was not verified for 15 years until 2005 [32], due to the difficulty in conductivity measurement of NAMs [43].

**Figure 3:**
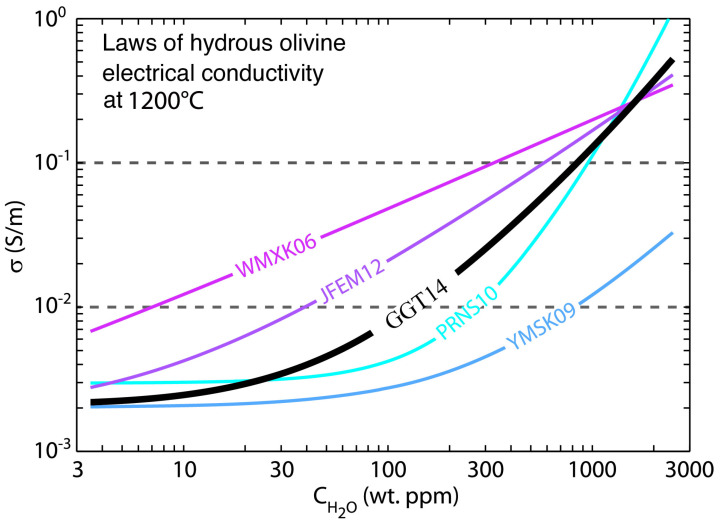
The effect of water on the conductivity of olivine at 1200 °C from various laboratory measurements (modified after Gardès et al. [58]). WMXK06:Wang et al. [54]; YMSK09: Yoshino et al. [45]; PRNS10: Poe et al. [56]; JFEM12: Jones et al. [59]; GGT14: Gardès et al. [58].

Nevertheless, there are discrepancies about the contribution of water on the electrical conductivity between different laboratory results. Here, we take the widely measured hydrous olivine for example. As shown in Figure 3, the effect of water on the electrical conductivity of olivine at 1200 °C from various laboratory measurements shows a large difference. Two endmember laws among these are from Wang et al. [54] and Yoshino et al. [45], separately representing that the conductivity is most sensitive and insensitive to the water content. The potential causes of this discrepancy are still in debate [45,57]. Therefore, it is important to keep in mind that the deduced water content from MT studies is largely dependent on the selected laboratory result. For example, to explain a conductivity of 0.01 S/m, the differences in the water content from two endmember models are over two orders of magnitude (Figure 3). Therefore, when determining the water content from MT, it is helpful to combine geological, geophysical and all other available information as we will discuss in Section 5.

In summary, there are two widely used formulas so far to fit the laboratory measurements on the conductivity of proton conduction [43,49], which can be expressed by one general equation:

(6) $$\sigma_{pro}=\sigma_{0p}\cdot C_{w}^{r}\cdot exp\left(-\frac{\Delta H_{p}-\alpha \cdot C_{w}^{1/3}}{RT}\right)$$

where $C_{w}$ is water content in weight wt%, and either α or *r* is a constant. These two equations are proposed due to the fact that the laboratory observations of hydrogen on conductivity mismatch the simple model by Karato [52]. To explain the deviations from this simple model, some studies use a value of 0.6–1 for *r* and zero for α [53,54,60,61,62,63], while others use a value of 1 for *r* and 1/3 for α [45,56,64,65].

Karato [66] proposed that there are multiple hydrogen species with various concentrations and degrees of mobility dissolved in the point defect of minerals, which can be described by the exponent factor *r*, whereas Yoshino [43] proposed that the charge transfer by proton hopping among point defects equally contributes to electrical conductivity i.e., r=1. For the geometrical factor α, Yoshino et al. [55,64] reported that the activation enthalpy for proton conduction depends on the water content and tends to decrease with increasing water content, which, however, was suggested to be negligible (i.e., α=0) [57,67].

Explaining the observed MT conductivity anomalies with the water content sometimes is challenging. Jones et al. [59] reported that neither the equation nor the related parameters discussed above can explain the observed MT conductivity anomaly in southern Africa using the water content derived from the xenoliths and hence proposed new parameters using a statistical method to satisfy the well-calibrated, high-quality field observations (Figure 3 purple line). It is, however, important to note that the water at the scale of xenoliths diffuses fast and thus may not represent the actual water content of the deep Earth [67,68].

In addition, by fitting all the available measured data in the laboratory, Gardès et al. [58] proposed a unified law on the electrical conductivity of hydrous olivine (Figure 3 black line). From the view of experiments, a law derived by accepting all of the measured data without discrimination may not necessary, in particular, because a few key processes that have heavily influenced the results were not shown by some experiments [57]. However, from the perspective of geophysics, the model that fits all the observed data within the data errors might be reasonable. Therefore, the model proposed by Gardès et al. [58] is also usually used in MT studies to infer the water content [69,70,71].

Similar to small polaron conduction, proton conduction is also affected by the main element chemistry, oxygen fugacity and temperature. As summarized by Karato [67], these influences on the conductivity of proton conduction are much smaller than the influence from water content, which could change the conductivity by a factor of 100–300 for the water content at a reasonable range of 10${}^{-5}$–1 wt% in the mantle. However, when considering other laws as shown in Figure 3, the influence of water content on the conductivity of proton conduction would decrease.

Hydrogen diffusion along the grain boundary is many orders of magnitude faster than that among the interior of lattice defects [32,72]. Based on previous experimental results, Selway [40] found that the effective increase in conductivity through grain boundary diffusion is only expected to occur for grain sizes smaller than fine-grained grains (10–100 μm), which is also supported by other studies [32]. However, the typical grain size in the upper mantle is on the order of 1 mm [72], and fine-grained grains only occur in particular regions such as in highly mylonitized and sheared zones and veins [32]. Therefore, hydrogen diffusion along the grain boundary is limited and unlikely to account for the large high-conductivity anomalies [40], which, in turn, could help to preserve water especially beneath the cratonic lithosphere as revealed by Yang et al. [73] in a previous MT study.

## 4. Mixing Models for Electrical Conductivity

Pure materials are uncommon, and general rocks of the crust and upper mantle are composed of several minerals and impurities. The bulk conductivity of rocks primarily depends on the conductivity, volume (percentage by volume, vol%), mass fraction (percentage by weight, wt%), and spatial distribution of each material, and can be calculated by mixing laws if this information is known. Alternatively, the electrical conductivity derived from MT studies is also bulk conductivity. Therefore, by combing the mixing models of electrical conductivity, MT results are able to constrain some important properties of the rock such as its porosity.

The most classic mixing law for two phases is Archie’s law Archie [74] which stands the test of time. The core assumption of this law is that there is only one conductive phase within a non-conductive phase. In this case, the bulk resistivity of a clean reservoir rock is controlled by the pore fluid while the contribution from the matrix that is much resistive can be negligible; and the other conductive phases such as the conductive clay minerals are nonexistent because these would provide an alternative pathway for the electrical current to flow [75]. It is important to note that this law was derived empirically but has been widely used successfully [75,76], which can be generally expressed as follows:

(7) $$\sigma =C\sigma_{f}\Phi^{-m}$$

where σ is the bulk conductivity, *C* is the empirical constant commonly close to 1 (see Glover et al. [75] for more detail), $\sigma_{f}$ is the conductivity of the fluid, Φ is the volume fraction of the fluid and m is the cementation factor determined by the connectivity of the fluid. Values of m in the range of 1–1.3 and ≥2 represent the fluids in well-connected and isolated pores, respectively.

While the initial usage of Archie’s law is for the water-saturated saline fluid, it is also suitable for the fluid of partial melt. The electrical measurement of partial melt olivine in the laboratory showed that at the low melt fraction (0.01–10 vol%) the bulk resistivity can be well described with C=1.47 and m=1.30 [77]. As shown in Section 3.2, the non-conductive silicate minerals would become semiconductors at high temperatures of the mantle and hence the contribution of the host rock to the bulk resistivity can not be negligible. Considering this, a modified Archie’s law was proposed [75] as follows:

(8) $$\begin{array}{c} \sigma =\sigma_{m}{\left(1-\Phi \right)}^{p}+\sigma_{f}\Phi^{m}\;,\\ \mathrm{with}\;p=\frac{\log(1-\Phi^{m})}{\log(1-\Phi )} \end{array}$$

where $\sigma_{m}$ is the conductivity of the solid matrix, and the exponents *m* and *p* separately describes the connectivity of the rock and fluid.

Another widely used two phase mixing model is the Hashin-Shtrikman bound model [78], which gives the upper (HS${}^{+}$) and lower (HS${}^{-}$) bound of the bulk conductivity:

(9) $$\begin{array}{c} \sigma_{HS}^{+}=\sigma_{f}\left(1-\frac{3\left(1-\Phi \right)\left(\sigma_{f}-\sigma_{m}\right)}{3\sigma_{f}-\Phi \left(\sigma_{f}-\sigma_{m}\right)}\right)\\ \sigma_{HS}^{-}=\sigma_{m}\left(1+\frac{3\Phi (\sigma_{f}-\sigma_{m})}{3\sigma_{m}+\left(1-\Phi \right)\left(\sigma_{f}-\sigma_{m}\right)}\right) \end{array}$$

The upper and lower bound represents the conductive material (e.g., fluid) in well-connected and isolated pores.

In a more general case, Glover [76] extended Archie’s law that allows for n phases as follows:

(10) $$\begin{array}{c} \sigma =\sum_{i=1}^{n}\sigma_{i}\Phi_{i}^{m_{i}},\\ \mathrm{with}\;m_{j}=\log\left(1-\sum_{i\neq j}\Phi_{i}^{m_{i}}\right)/\log\left(1-\sum_{i\neq j}\Phi_{i}\right) \end{array}$$

and Berryman [79] extended the Hashin–Shtrikman bound model to a model that allows for n phases as follows:

(11) $$\begin{array}{c} \sigma_{HS}^{+}={\left(\sum_{i=1}^{n}\frac{\Phi_{i}}{\sigma_{i}+2\sigma_{max}}\right)}^{-1}-2\sigma_{max}\\ \sigma_{HS}^{-}={\left(\sum_{i=1}^{n}\frac{\Phi_{i}}{\sigma_{i}+2\sigma_{min}}\right)}^{-1}-2\sigma_{min.} \end{array}$$

## 5. Causes of High Conductivity

**Figure 4:**
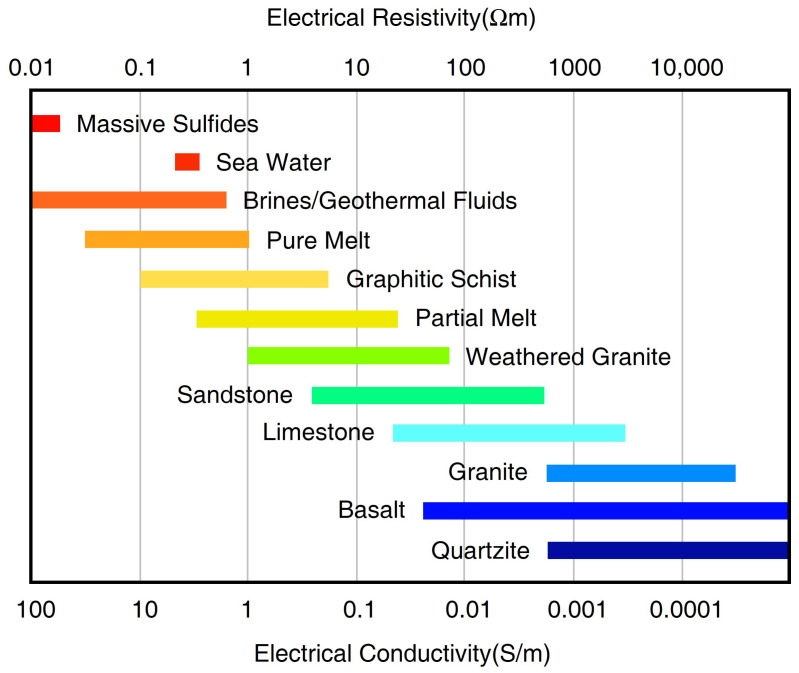
The electrical resistivity (or its inverse conductivity) values for various rock-forming materials by Comeau [85]; the values were taken from Simpson and Bahr [1] and references therein.

During the past few decades, a large number of MT studies were successfully conducted and comprehensively-reviewed [80,81,82]. Among these studies, the most common high-conductive candidates to interpret the observed high-conductivity anomalies include saline fluids, partial melts, grain-boundary graphite films, sulfides, and water (hydrogen) in NAMs (Figure 4). As this review is mainly for the deep crust and mantle, MT studies in ore exploration that were previously well reviewed [83,84], are not discussed here. It is important to note that the conductive materials must form interconnected networks within the resistive rock matrix in order to enhance bulk conductivity.

### 5.1. Saline Fluids

Free saline fluids are typically found in highly porous sedimentary rocks, fractured and sheared zones within the brittle mid-upper crust, and dehydrated regions. In the sedimentary basin (Figure 1), the near-surface high-conductivity anomalies are generally caused by the saline fluids that interconnect through a high porosity network [8]. Additionally, clay minerals in sediments can adsorb cations to their surfaces to form an electric double layer, which further enhances the conductivity of the sediments [5]. In particular, highly conductive smectite (<10 Ω.m) formed during hydrothermal alteration often occur as caps in volcanic geothermal areas, and with increasing depth transform to less conductive illite (10–60 Ω.m) [86]. These features were well imaged by related MT studies [18,87].

Well logging to the depth of 12.3 km in the Kola Peninsula of Russia and 8.9 km in Bavaria of southern Germany demonstrated that the high concentrations of saline fluids are stable at least at mid-crustal depths [1]. In tectonically stable regions, fluids that are unlikely to flow downward extend into the ductile regions that contain little interconnectivity of the porosity due to increased pressure; even in regions with low geothermal gradient, where the brittle-ductile transition could occur in the lower crust, these fluids that reach the stable lower crust will be used in mineral reactions [40,88]. In addition, following the cessation of tectonic activity, the residence time of these fluids was considered to be on the order of 100 Ma [89,90,91,92]. This is very short in geological terms and hence interconnected saline fluids are generally unlikely to account for the observed high-conductivity anomalies in tectonically stable cratonic regions [40,93,94,95].

**Figure 5:**
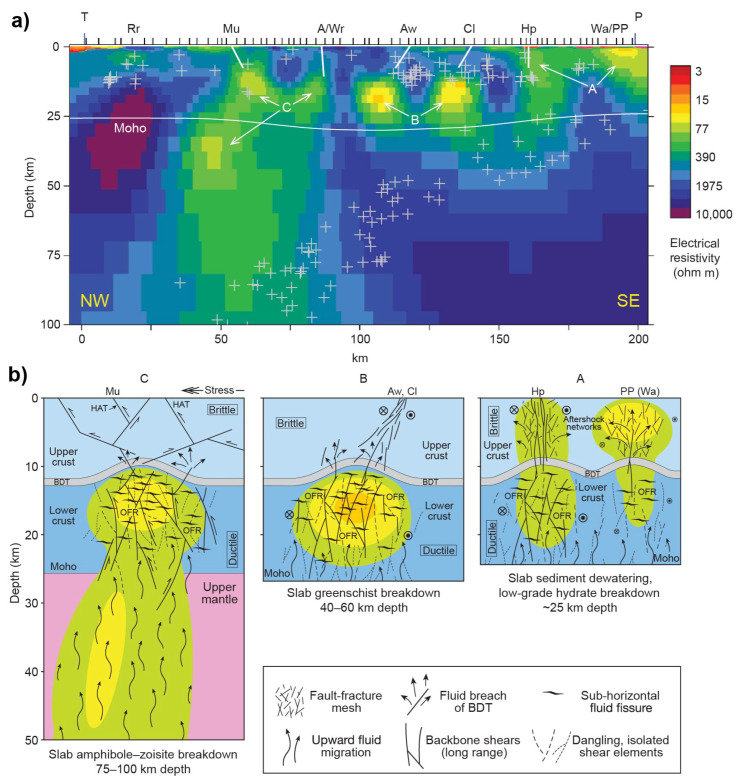
(**a**) MT derived electrical resistivity structure of an advancing subduction system at Marlborough, New Zealand and (**b**) related interpretation of the high-conductivity anomalies after Wannamaker et al. [96]. P denotes the Pacific ocean. Hope (Hp), Clarence (Cl), Awatere (Aw), and Alpine/Wairau (A/Wr) are four strike-slip faults; Murchison (Mu) represents a thrust fault. Plus symbols denote seismicity within 25 km along the MT profile. BDT = brittle–ductile transition; OFR = overpressured fluid reservoir; HAT = high-angle thrust. Green, yellow and orange zones in (**b**) represent the low resistivity zones as imaged in (**a**) NW: Northwest; SE: Southeast. A, B and C represents the high conductive zone.

In contrast, saline fluids likely enhance electrical conductivity in tectonically active regions, where the deformation improves fractures and/or faults such as in subduction or rift zones. Wannamaker et al. [96] presented an excellent example of the saline fluid and deformation regime of a transpressional advancing subduction system (Pacific plate) beneath the Marlborough strike–slip fault system of New Zealand. As shown in Figure 5, they proposed that the conductors (labeled A, B, and C) are caused by the interconnected saline fluids from various dehydration mechanisms at different depths along the interface of the subducted plate. Interconnection of fluids is ensured by the fault-fracture meshes in the brittle upper crust, and by the long-range backbone shears in the ductile lower crust benefiting from the transpressional deformation [96,97]. The deep fluids generated from dehydration reactions or dewatering would migrate upward to the crust due to their lower density, get trapped below the brittle-ductile transition area and remain there [98] or occasionally breach it induced by fault-valve or other tectonic events [96,97,99]. In addition, a recent study using MT and control-source EM revealed the importance of fluid-rich subduction topography such as seamounts on the generation of forearc porosity and associated fluid transportation [37]. Apart from oceanic subduction, continental subduction can also transport water into the interior of the Earth [100]. Zhang et al. [101] observed a large-scale crustal conductor beneath Altyn Tagh Range, the northern margin of the Tibetan Plateau, which was interpreted as saline fluids originating from prograde metamorphic reactions of the underthrusting Tarim Block under high temperatures and high pressure.

**Figure 6:**
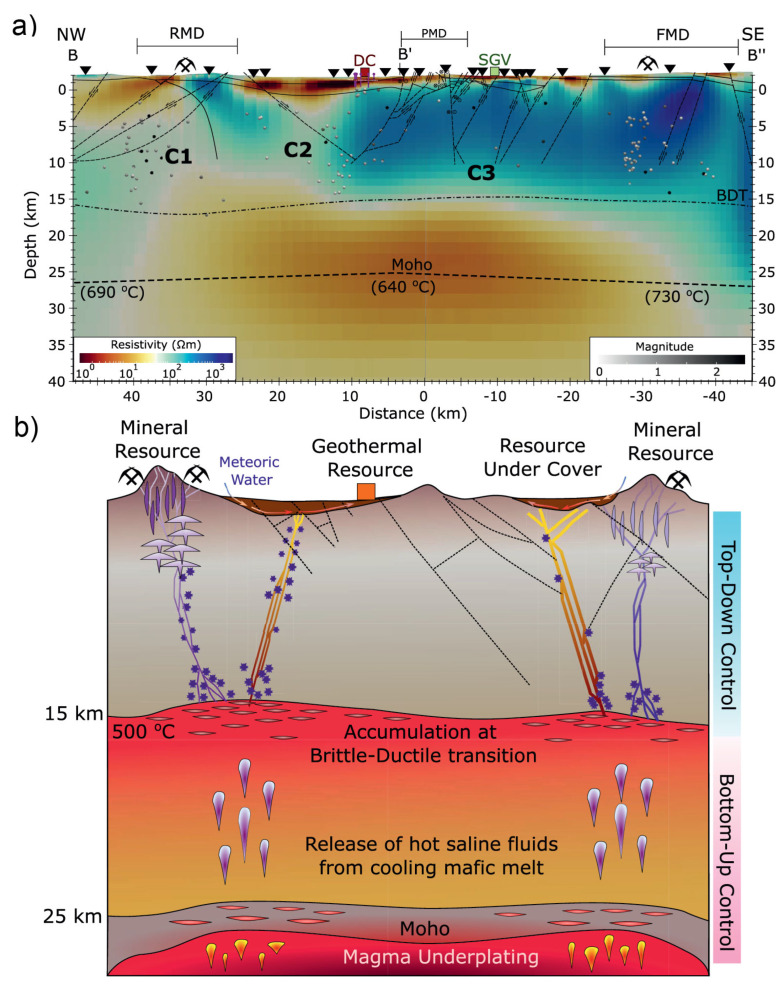
(**a**) A transect of electrical resistivity model extracted from 3D MT inversion at Gabbs Valley in the Great Basin, western United States and (**b**) the corresponding conceptual model after Peacock and Siler [34]. Dashed lines in the upper crust are faults inferred from structural geological data and spheres represent earthquake locations. C means conductor. RMD = Rawhide mining district; PMD = Poinsettia mining district; FMD = Fairplay mining district; DC = Don A. Campbell geothermal power plant; SGV = Southeastern Gabbs Valley geothermal area; BDT = brittle–ductile transition. Dashed lines in the upper crust are faults, squares are hydrothermal systems, triangles are MT sites, and pick and hammer are mineral systems.

Moreover, fractures and/or faults often form in zones of extensional tectonics. One of the recent prominent examples found the control of deep fluid sources on the near-surface geothermal and mineral resources in the Gabbs Valley area of Basin and Range Province [34]. As shown in Figure 6, they proposed a conceptual model to interpret the obtained electrical resistivity structure from 3D MT inversion. In this model, the observed high-conductivity anomalies below 15 km reflect the modern magma underplating and hydrothermal fluid release, which rise buoyantly and get trapped below the brittle-ductile transition forming conductive anomalies and inducing overpressured zone that episodically breaches the brittle-ductile transition as a reslult of weak tectonic events [34,102]. In brittle regimes, fluid transportation-related conductive features through the fault and fracture meshes are controlled by Walker Lane and Basin and Range tectonics [34].

Generally, the resistivity of saline fluids is in the range of 0.01–1 Ω.m (Figure 4) and is controlled by salinity, temperature and pressure. An increase in temperature would increase the mobility of charge carriers and hence enhance the conductivity Equation (4), nevertheless at high temperatures above 300 °C, the conductivity of crustal fluids tends to be constant or even decrease due to the decreasing viscosity of water [103]. The increasing pressure has little influence on fluid conductivity at lower temperatures below 300 °C, while above this temperature, it can increase the conductivity by increasing the solution density [104]. Apart from the generally discussed chloride salts, the bicarbonate (containing HCO${}_{3}^{-}$) was proposed to dominate the conductivity at greater depths in lithostatic systems Nesbitt [103]. However, experimental studies demonstrated the dihedral angles (see Evans [5] for more details) of H${}_{2}$O–CO${}_{2}$ fluids would be too large to maintain interconnection at greater depths [105]; hence the fluids containing a large amount of CO${}_{2}$ are unlikely to be interconnected to enhance the conductivity at mid and lower crust [104].

The volume fraction of saline fluid or porosity of a fluid-saturated rock can be estimated in MT studies combining the mixing laws shown in Section 4 and a variety of electrical conductivity measurements of NaCl–bearing aqueous fluids at high temperature and high-pressure [103,106,107]). Other types of studies such as petrological and geochemical studies can provide valuable information on parameters such as salinity that determine the resistivity of the saline fluids in a specific study area [26,87]. If unknown, the salinity and/or interconnection of fluids especially for the deep mid-lower crust, can sometimes be assumed or the end-number can be tested in order to constrain fluid contents [96,108,109,110]. The end-member model was achieved by assuming the resistivity of general 0.01–1 Ω.m and/or various interconnection (cementation factor in the ranges of 1–2) for saline fluid. Recently, Hu et al. [111] measured the electrical conductivity of the dry and hydrous (hydrogen in point defects) plagioclase (a major rock-forming mineral in the crust) at high temperature and pressure, and found that this cannot account for the general high-conductivity anomalies observed in various MT studies in the mid-lower crust, whereas at least 1 vol% of fluid with a salinity of 3.5 wt% would be necessary. Therefore, significant influences on enhanced conductivity are likely from the interconnected saline fluids or other high-conductivity phases instead of the rock matrix, making MT suitable to constrain the fluid fraction.

### 5.2. Partial Melting

In addition to the saline fluid, partial melting is another strong candidate responsible for the high-conductivity anomalies observed in MT surveys [104,112,113]. Partial melting occurs through three main events, namely a decrease in pressure, an increase in temperature, and the addition of volatiles such as water; therefore, it generally occurs in tectonically active regions such as mid-ocean ridges [35], subduction zones [114,115] and volcanic regions [26,116], but unlikely to occur in tectonically stable cratonic regions [40]. In particular, the addition of water has the potential to lower the solidus of the rock, and from dry to water-saturated rocks, and the initial melting temperature typically reduces from around 1200 °C to 650 °C [112,117]. As discussed above, fluid could exist throughout the crust column and even in the upper mantle in tectonically active regions; therefore, water is a potential factor to trigger partial melting in areas under this temperature range such as in the lower crust.

The earth’s crust and upper mantle are generally composed of silicate minerals, making them the strongest candidate for partial melts; nevertheless, carbonatite and sulfide melts are also possible. In particular, molten carbonates have much higher conductivity than the molten silicates of at least two orders of magnitude according to the laboratory measurements at high temperature and high-pressure [118,119], and hence much lower amount of melting would be required than silicate melts to explain the high-conductivity based on the mixing models in Section 4. However, as carbonate melts are stable only at depths below 75 km (>2.5 GPa), as shown in experimental petrology studies [120], and are very rare in surface samples, it is not clear how common they are in the mantle [5], and highly fortuitous conditions are required for the presence of carbonate melts to account for high-conductivity as suggested by Karato [67]. If present at great depths, carbonatite melts are expected to migrate upward; when both carbonatite and silicate melts are present, they are immiscible and silicate melts would dominate the interconnection, while carbonatite melts would likely not be well connected [121,122], and therefore the bulk resistivity of melts would be attributed to the silicate melts [5]. In contrast, when both sulfides melt and basaltic melts are present, sulfide melts would enhance the bulk conductivity of the melts and reduce the partial melt fraction required to interpret the observed high-conductivity as proposed by Ducea and Park [123] based on the petrographic studies of xenoliths. However, sulfide melts were barely used to account for large-scale high-conductivity considering its small concentrations typically 0.7 wt% for the Earth [124] and 0.06 wt% for the mantle [125].

**Figure 7:**
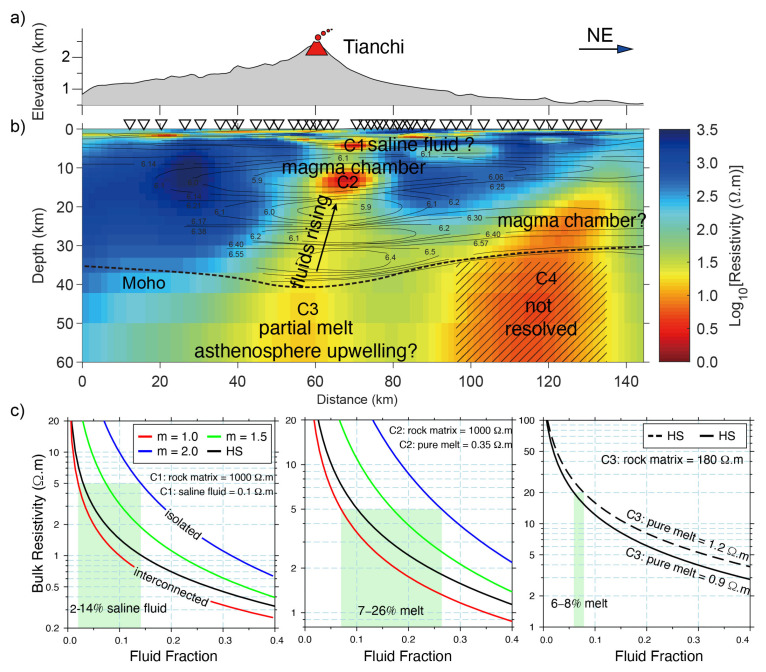
(**a**–**c**) MT resistivity model of Changbaishan-Tianchi volcano system and its implications for fluid fraction modified after Yang et al. [26].

**Figure 8:**
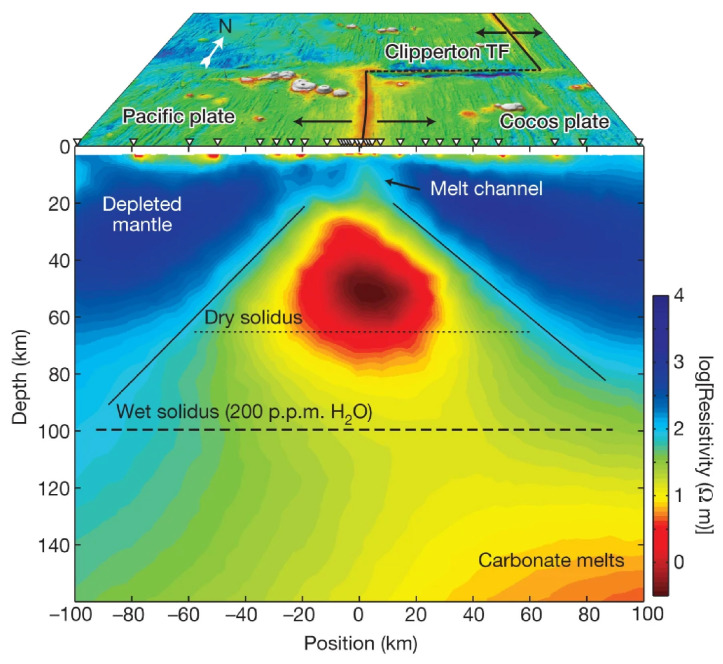
MT resistivity model beneath the east Pacific Rise and the related interpretation after Key et al. [35].

As shown in Figure 6, the upper mantle with high-conductivity observed in the Basin Ridge region of the western United States was interpreted as basaltic melts generated by mafic underplating [34,102]. A similar upper mantle high-conductivity anomaly beneath the Changbaishan-Tianchi volcano was also explained by silicate partial melting that might be caused by the decompression melting of the upwelling asthenosphere (Figure 7) [26]. Carbonate melting seems to more generally occur in the oceanic plate [35] or subduction-related regions [126] at great depth (>75 km) [120] to interpret the high-conductivity anomaly. As shown in Figure 8, Key et al. [35] obtained the electrical structure of the East Pacific Rise region from seafloor MT data and interpreted the observed high-conductivity as partial melting of the upwelling mantle. They found that the upwelling caused dry and wet peridotite melting at depths of 65 and 100 km, respectively, and interpreted the high conductivity at greater depths (140 km) as carbonate melt. These deeper carbonate melts should migrate upward and increase the conductivity at the depth of around 100 km, which however exhibits relatively low conductivity. This is consistent with the experiments that the silicate melts would dominate the interconnection and thus lower the conductivity when silicate and carbonate melts are simultaneously presented as we discussed above [35,120].

### 5.3. Grain-Boundary Graphite Films

Interconnected graphite films along grain boundaries are often used to explain the high-conductivity anomalies observed in the continental crust and lithospheric mantle [40,93,127,128]. The carbon-forming graphite may come from the transformation of organic-rich sediments [129] or precipitated from fluids produced by the mantle magma or the metamorphism of carbonate rocks [39]. These are all important sources for the formation of graphite films.

**Figure 9:**
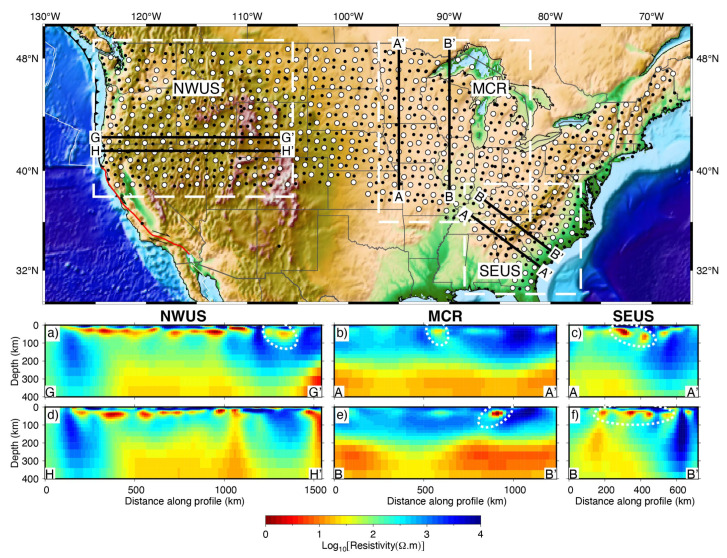
MT resistivity models derived from USArray MT data in the northwestern United States (NWUS) [114], the Midcontinental Rift (MCR) [94] and the southeastern United States (SEUS) [132] (modified after Yang et al. [73]). In the upper panel, the white dashed lines denote the areas of each study; black lines denote profiles discussed in these publications, labeled as in the original publications. The dots denote the MT data. (**a**–**f**) The areas surrounded by white dashed lines are interpreted as graphitic or sulfide-bearing metasediments that are deeply trapped along the ancient collision belts.

The formation of graphite generally requires high temperatures (600–2000 °C), while high strain energy could promote the graphitization, allowing the graphite to form at lower temperatures (400–500 °C) [1,130,131]. The increase in pressure could contribute to the connectivity of the graphite network [1]. Hence the high conductivity observed in regions of extensive deformation (e.g., crustal shear zones) was generally attributed to the interconnected graphite film [5,92]. Similarly, the high conductivity along the suture zone as well as in the fold-thrust region may also be associated with graphite. For instance, elongated high conductors, often occurring in the suture zone, were interpreted to the graphite/sulfide-rich sediments that were trapped by underthrusting along the ancient collision belts (Figure 9) [94,114,127,132]. Low-pressure environments, at very shallow depths, such as local uplift may disrupt the connectivity of graphite films, resulting in a decrease in electrical conductivity [133]; however, the graphite could reinterconnect when the pressure increases to a certain level, as has been observed in rock samples [134]. Therefore, the pressure-induced graphite reconnection can cause highly resistive rocks to behave as conductors at great depth and vise versa [1], which might be a useful indicator of geological evolution.

It has been shown that graphite films are stable at limited depths [1]. It will be destroyed at temperatures greater than 737 °C [39] or 900 °C [40] and will transform into highly resistive diamond (900–1300 °C and 45–60 kPa) through phase transitions [1]. Thus, graphite is generally not used to explain the high-conductivity anomalies in the upper mantle at depths greater than 150–200 km [1,40]. The stability of graphite is also influenced by its thickness and volume fraction. Experimental studies found that graphite films with grain boundaries less than 100 nm decrease rapidly at temperatures greater than 727 °C [39]. However, they may remain connected for long geological times if the thickness of graphite films is on the millimeter level. Under the temperature conditions of the lithospheric mantle, the connected graphite film will become stable when the graphite volume fraction is small (less than 1%) [135], while the graphite content is high, the graphite layer will be stable and become the cause of high-conductivity in the mantle [95,128]. In addition, graphite (and diamond) is unstable under strong oxidation conditions and will be converted to carbonate minerals at temperatures below solidus and to carbon-rich melts at temperatures above solidus [40], while under strong reduction conditions, graphite may precipitate out of carbon dioxide or hydrocarbon-rich metamorphic fluids to form graphite films [136]. Therefore, graphite films are usually not the cause of high-conductivity anomalies under strong oxidation conditions.

Graphite as the conductive phase in the lower crust is still under debate. Yardley and Valley [88] argued that graphite tends to form isolated disconnected sheets in high-grade metamorphic formations in the lower crust and thus cannot produce observable high-conductivity anomalies. However, it has been suggested that high-grade metamorphic rocks containing isolated graphite can form interconnected graphitic veins with high conductivity through retrograde metamorphism at reduced temperatures based on deep drilling results [90,129,137]. In addition to the retrograde metamorphic fluids described above, carbon-rich magmatic fluids can also precipitate graphite in the lower crust [129] and be interconnected through subsequent shearing, thereby forming a highly conductive lower crust [95].

In general, a small amount of interconnected graphite can significantly enhance bulk conductivity, but evidence for the presence of large regions of interconnected graphite is still limited [5]. Geological evolution has a dramatic impact on the presence or absence of graphite films, and geochemical depletion or enrichment of lithosphere associated with tectonic and kinetic events (especially in the recent past) often correspond to the destruction or presence of graphite films, respectively [40].

### 5.4. Sulfides

Sulfides as the causes of conductive anomalies have often been discussed together with graphite. The observed high conductivity in the lower crust and/or upper mantle at terrane boundaries, suture zones, subduction zones, or orogenic zones is often interpreted as the deeply trapped graphitic or sulfide-bearing metasediments (Figure 9) [94,114,127,132,138]. The difference is that carbon-rich fluids, especially carbon dioxide, are prevalent in high-grade metamorphic strata and are important constituents of volatiles released by magmatic activity [39], whereas sulfides are generally small in volume [33,40], and 0.7 wt% in the Earth [124], about 0.06 wt% in the mantle [125,139]. Therefore, the anomalies caused by sulfide minerals are usually small. Furthermore, sulfide is usually unstable below the lithosphere [40] and unlikely to generate extended conductive anomalies in the mantle [33].

Therefore, the interpretation of observed high-conductivity in terms of sulfides often requires evidence of its occurrence in the study area. For instance, Jones et al. [140] suggested that interconnected pyrite sulfides in North American Central Plain were responsible for the formation of high-conductivity based on the analysis of rock samples containing sulfides. By contrast, the sulfides were ruled out as the cause of high conductivity due to the absence of sulfide in the mantle xenolith of the Slave craton [93]. Some metal deposits closely associated with sulfides could provide indirect evidence of the existence of sulfides. For example, high-conductivity anomalies in the crust were interpreted as sulfides due to the presence of gold ores in different studies [110,141]. In addition, the reduction environment generated by the interaction between mantle-derived magma and the crust is conducive to the precipitation and accumulation of sulfides in the lower crust [95,139,142]; the plutonism may also help to form the interconnected sulfides along the intrusive paths of the magma [5], both of which could attribute to the high-conductivity.

**Figure 10:**
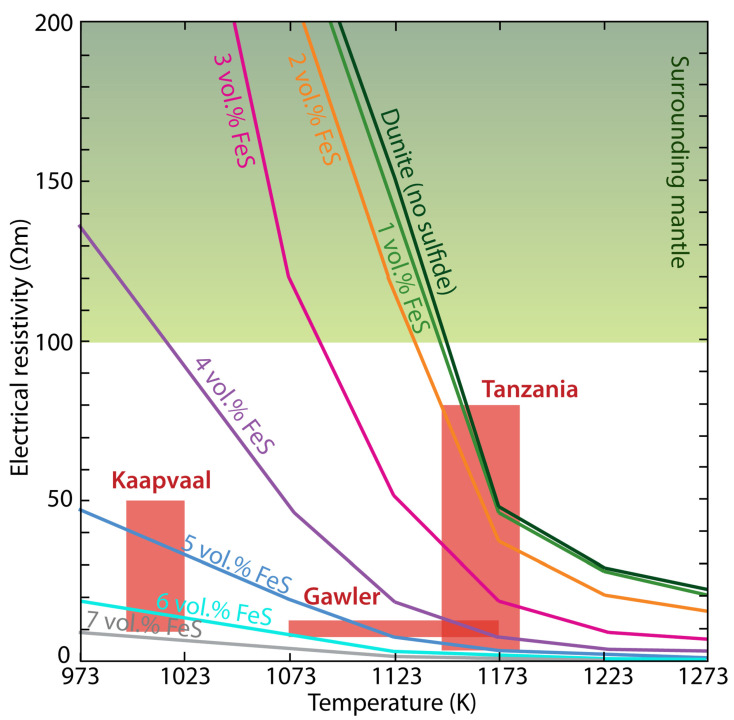
The sulfide content required to interpret the observed high-conductivity at different cratonic contexts according to the electrical laboratory measurements [143].

Recent electrical laboratory measurements of xenoliths containing sulfides from several cratons reveal that a few vol.% sulfides can be used to explain their high-conductivity anomalies (Figure 10), as indicated by Saxena et al. [143]. However, a much lower content of sulfides could explain these anomalies if the mixing models in Section 4 were used as the resistivity of the sulfides is very low. The discrepancy between the measurement and the calculation is caused by the fact that the sulfides were not well connected according to the measurement [143], while fully connected might be assumed by calculation. Similar caution should be paid to the graphite content estimate due to the lack of related electrical laboratory measurements. In contrast, the content estimate of saline fluids and/or melts from electrical conductivity is generally more accurate due to extensive experiments as summarized by Pommier and Le-Trong [144], Pommier and Roberts [145]. Therefore, qualitative or semi-quantitative interpretations may still predominate for the graphite and/or sulfide [92,146], while semi-quantitative and even quantitative interpretations become more and more general for the saline fluids and/or melts [26].

### 5.5. Hydrogen in Nominally Anhydrous Minerals

Temperature, water (hydrogen in NAMs), and partial melting are the dominant factors controlling the rheology of the upper mantle [147]. It has been shown that a small amount of water in the mantle can affect the melting and rheology of the mantle, and therefore the water distribution in the mantle has a significant influence on the evolution and dynamics of the Earth [67]. Geophysical methods can be used to constrain the spatial distribution of water content, among which electrical conductivity imaging is the best method because it is highly sensitive to water content and less influenced by other factors such as temperature, oxygen fugacity, and major chemical elements [57,59,67].

The primary mineral making up the upper mantle is olivine, whose conductivity has been extensively studied in mantle conditions. By combining these laboratory measurements with the electrical structure derived by MT, the water content and/or melt fraction can be inferred. [15,148]. A comparison of the conductivity model for dry olivine given by Constable [44], which takes into account temperature-dependent small polaron and magnesium vacancy conduction, with the MT results allows determining whether other high-conductivity phases are needed [102,148]. When a high-conductivity phase is required, the hydrogen content of olivine is often calculated to try to explain the observed high-conductivity, considering that olivine is the dominant mineral of the upper mantle and a large number of conductivity experimental results with hydrous olivine [45,54,61]. The water content required to produce the high-conductivity anomaly can be constrained by comparing the conductivity of olivine at different temperatures and water contents and that obtained from MT inversion. For instance, Meqbel et al. [114] used the experimental results of Poe et al. [56] to estimate the water content of the upper mantle in the northwestern United States; Yang et al. [94] selected the peridotite model of Dai and Karato [149] to infer the water content of the upper mantle in the northeastern United States.

However, as mentioned above, the conductivity models obtained by different research groups under the same temperature and water content conditions are not consistent and even widely vary, which affects the accurate estimation of water content. For example, by measuring the conductivity of hydrous wadsleyite and ringwoodite, the main minerals in the mantle transition zone (MTZ), Huang et al. [53] found that the conductivity of these minerals is very sensitive to the variation in the water content. They suggested that the MTZ is hydrous and about 0.1–0.2 wt% of water content can explain the conductivity of the MTZ observed by Utada et al. [150] in the North Pacific (about 0.1–0.5 S/m) [53]. However, [55] supposed that the MTZ is anhydrous and the dry MTZ can well explain the depth-conductivity profile (about 0.02–0.2 S/m) obtained by Kuvshinov et al. [151] for the North Pacific. It is notable that Yoshino et al. [55] did not exclude the possibility of less than 0.1 wt% water because they suggested that the contribution of proton conduction is relatively small at high temperatures and that the conductivity of dry minerals and water containing 0.1 wt% are almost equal in the MTZ.

**Figure 11:**
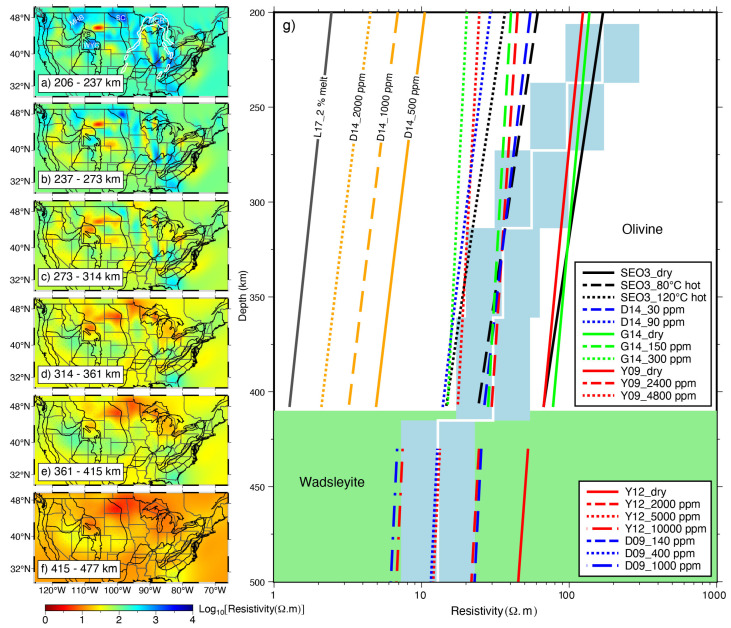
MT resistivity model of the continental United States and its implications for water content or temperature modified after Yang et al. [73]. SEO3: Constable [44]; D14: Dai and Karato [61]; G14: Gardès et al. [58]; Y09: Yoshino et al. [45]; Y12: Yoshino and Katsura [65]; D09: Dai and Karato [60]; L17: Laumonier et al. [153].

Therefore, multiple experimental models can be considered [132], or a possible range of water content can be given in combination with existing end-member models (models that predict the highest resistivity and conductivity) in practical interpretations [110,148]. As shown in Figure 11, to determine the water content, Yang et al. [73] took into account both end-member laboratory models and a comprehensive model that takes into account all available laboratory results, and thus obtained a relatively reasonable water content of the United States at depths of 200 km to mantle transition zone. In addition, both the maximum solubility of water under specific temperature and pressure conditions [152] and whether the inferred water content will induce partial melt [147,148] should also be fully considered, in case unreasonable water content was estimated by the MT studies.

Simplifying the material composition of the upper mantle makes the calculation of water content relatively easy, but this simplification may introduce bias in the estimation o water content [82]. Considering a composition model close to the real mantle may improve the accuracy of conductivity anomaly interpretation to some extent [40,48,82]. High-temperature and high-pressure experiments on the effect of water on the conductivity of other NAMs that constitute the mantle are relatively few in comparison to olivine, and the models obtained from different groups are also diverse [15,16,154]. Moreover, in the case of a multi-mineral system, the partition coefficients between different minerals in addition to their solubility are also needed to be considered [154], although the partition coefficients obtained from different experiments are also various [48,147]. In general, taking into account the actual mantle composition also presents a number of challenges, such as the vast discrepancies in the conductivity obtained using the end-member model when the water content and mantle rocks are both constant. Consequently, there is much ambiguity and dependence on the choice of laboratory model when estimating the water content from MT data [82].

When the upper mantle observed by MT is highly resistive, the temperature will be the most important factor controlling its conductivity [50]. In this case, MT results combined with the experimental model of dry mantle minerals, lithologically constrained mineral fraction, as well as the mixing models (e.g., HS${}^{-}$ bound), can be used to derive the temperature distribution of the mantle [51]. It was found that the difference between the conductivity obtained by the HS upper and lower bound is small for the main mineral assemblages of the mantle in the absence of other interconnected conductive phases [51,155], and thus the lack of mantle mineral composition constraints may not significantly affect the estimation of the temperature distribution for a particular study area [82]. However, Naif et al. [82] found that the MT method is limited to constrain absolute resistivity values for ultra-high resistances greater than 10${}^{4}$ Ω.m through various one-dimensional forward modeling tests, which limits the estimation of mantle temperature that less than about 850 °C (without highly conductive sedimentary layers) or 1000 °C.

## 6. Conclusions

The high-conductivity anomalies observed in MT are influenced by various factors, mainly including temperature, composition, saline fluids, partial melting, graphite, metal sulfides, and hydrogen content in nominally anhydrous minerals. In this paper, the conduction mechanisms, influencing factors, and relationships to the tectonic evolution of each of the conductive phases were systematically discussed.

Overall, very small amounts of connected fluids and/or partial melts can strongly influence bulk conductivity. They are often found in tectonically active areas such as continental collision zones [30,31,156], subduction zones [96,157,158], volcanic zones [159], and rift zones [35], while in tectonically stable regions they are unlikely to be the cause of high-conductivity anomalies [40]. Graphite and/or metal sulfides are commonly used to explain the high conductivity observed in suture zones or terrane boundaries, where they occur in the lower crust or even the upper mantle through subduction and/or orogeny [94,114,127,132,138]. However, it is important to note that sulfide has a small volumetric content and can be ruled out without existing evidence [40]. In addition, the temperature and pressure conditions need to be considered for the presence of graphite films [40]. Crustal metal mineralization and clay minerals in sedimentary basins can also cause high-conductivity anomalies. Hydrogen content in NAMs can significantly increase the conductivity of the lithospheric mantle and the lower crust. High-temperature tectonothermal events can decrease the hydrogen content and destroy the interconnected graphitic films, while interactions with fluids from subduction slabs or mantle plumes can enrich hydrogen as well as graphite of the lithosphere [40], and thus increase its conductivity. Therefore, the geological setting and evolution history of the study area needs to be fully considered when interpreting the observed highly conductive structures by MT.

The MT method is able to provide semi-quantitative as well as quantitative constraints on various information such as saline fluid fraction, partial melt fraction, hydrogen content, and temperature of the mantle by combining petrology, laboratory experimental results, and mixing models. In contrast, qualitative or semi-quantitative interpretations may still predominate for the graphite films and/or sulfides. It is also important to note that large uncertainties still exist for the semi-quantitative and quantitative interpretation, especially in the estimation of hydrogen content in NAMs. When considering the real mantle composition for the water content estimation, the selection of mantle rocks, the conductivity measurements and partition coefficients of each mineral, and the mixing models probably are the main sources of uncertainty. Therefore, it is necessary to fully integrate the lithological, seismic, geochemical, and all other available results to reduce the uncertainty in the interpretation of electrical conductivity anomalies derived from MT studies; thus, this area of study significantly contributes to the exploration of deep earth, deep sea and even extraterrestrial planet in the future.

## Data Availability

No data was used in this study.
